# A Case Report of Refractory Diffuse Alveolar Hemorrhage as a Complication of Antiphospholipid Syndrome and Review of Its Management

**DOI:** 10.7759/cureus.47267

**Published:** 2023-10-18

**Authors:** Denis Ruzdija, Aaron Bertolo, Shaunak K Pandya, Akshay Amaraneni

**Affiliations:** 1 Internal Medicine, University of Arizona, Tucson, USA; 2 Hematology and Oncology, University of Arizona, Tucson, USA; 3 Hematology and Oncology, University of Arizona Cancer Center, Tucson, USA

**Keywords:** immunosuppressive therapy, dah, aps, antiphospholipid syndrome, diffuse alveolar hemorrhage

## Abstract

Diffuse alveolar hemorrhage is a rare complication of antiphospholipid syndrome with high mortality rates. Early diagnosis and treatment with steroids and immunosuppressive agents, along with the achievement of hemostasis and hemodynamic stability, is critical to improving outcomes. This case demonstrates the complexity of managing refractory diffuse alveolar hemorrhage in a 48-year-old woman with antiphospholipid syndrome requiring treatment with high-dose corticosteroids, rituximab, and cyclophosphamide.

## Introduction

Antiphospholipid syndrome (APS) is a rare condition associated with prothrombotic and pregnancy-related complications [1]. APS has an estimated prevalence of 40-50 cases per 100,000 individuals [2]. APS can affect multiple organ systems including hematologic (e.g., arterial or venous thrombosis, thrombocytopenia), cardiac (e.g., myocardial infarction, valvular dysfunction), pulmonary (e.g., pulmonary embolism, pulmonary hypertension, hemorrhage), dermatologic (e.g., rash, skin ulcers/lesions), renal (e.g., glomerular injury, infarctions), neurological (e.g., stroke, seizures), and others [3]. The pathogenesis of APS involves autoantibodies targeting β2-glycoprotein I, which is a plasma protein with an affinity for phospholipids, present on endothelial surfaces. The binding of autoantibodies to β2-glycoprotein I leads to an increased expression of prothrombotic cellular adhesion molecules, as well as a decrease in proteins that aid in anticoagulation [4].

Diagnosis of APS is based on the revised Sapporo criteria which includes both clinical and laboratory findings [5]. Diagnosis is based on the presence of a persistent antibody and at least one of the clinical criteria. Clinical criteria include one or more events of vascular thrombosis (arterial, venous, or small-vessel) or pregnancy complications such as one or more unexplained loss of morphologically normal fetus at or beyond 10 weeks; at least one premature birth of a morphologically normal infant before 34 weeks due to eclampsia, pre-eclampsia, placental insufficiency; or at least three spontaneous, consecutive abortions before 10 weeks without an explanation or cause. Laboratory criteria include the presence of any of the following markers at least 12 weeks apart: lupus anticoagulant, anticardiolipin antibody (IgG or IgM) present in medium or high titers (>40 GPL or MPL or >99th percentile), or anti-β2-glycoprotein I antibody (IgG or IgM) >99th percentile.

Treatment of APS requires long-term anticoagulation with a vitamin K antagonist, such as warfarin [6]. During pregnancy, the treatment often requires low-molecular-weight heparin along with aspirin. Although treatment with warfarin can be effective, APS can be very challenging to manage in those with pulmonary complications. In addition to pulmonary embolism and infarct, the formation of micro-thrombi in pulmonary vasculature can often lead to pulmonary hypertension; in rare cases, it can lead to pulmonary capillaritis, bland alveolar hemorrhage, or diffuse alveolar hemorrhage [7]. Management of diffuse alveolar hemorrhage is especially difficult as patients are often already receiving anticoagulation with warfarin. Here, we describe a case of refractory diffuse alveolar hemorrhage in a patient with APS and review its management.

## Case presentation

**Figure 1:**
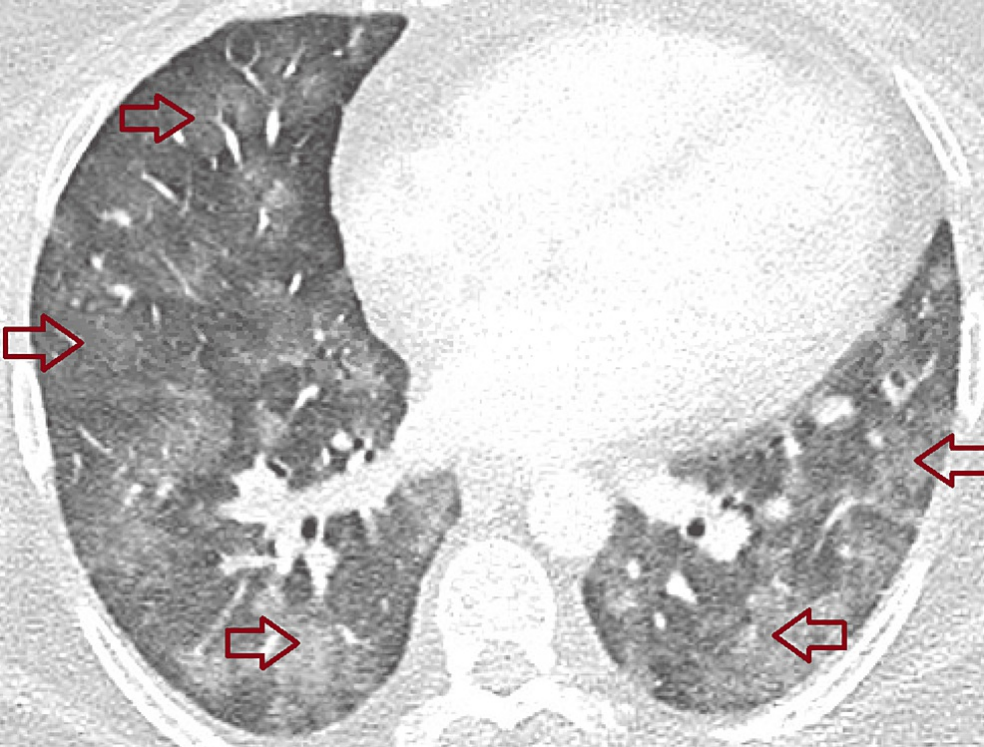
CT of the chest demonstrating multiple bilateral ground-glass opacities (red arrows).

**Figure 2:**
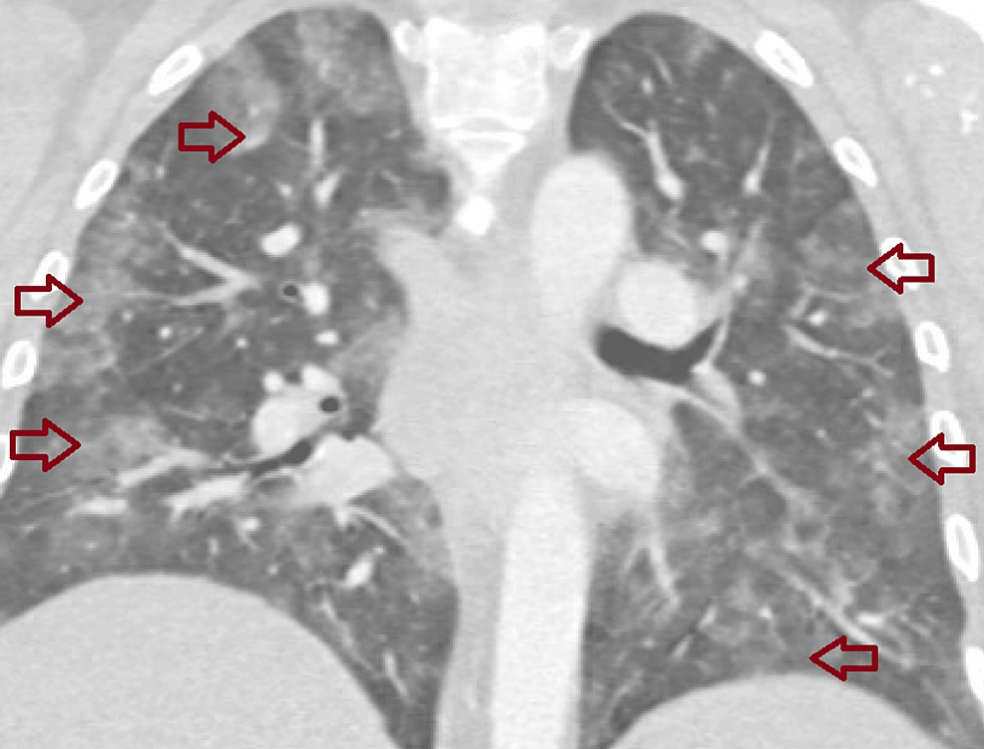
CT of the chest demonstrating multiple patchy bilateral peripheral and basilar ground-glass opacities (red arrows).

A 48-year-old woman with chronic hypoxic respiratory failure due to pulmonary hypertension (requiring 4 L of oxygen via a nasal cannula at baseline) and APS, diagnosed in 2016 after right middle cerebral artery ischemic infarct, presented with recurrent hemoptysis and increased dyspnea while on chronic anticoagulation with warfarin. Of note, she was previously diagnosed with diffuse alveolar hemorrhage in 2019 requiring mechanical ventilation and was treated with rituximab at that time. On presentation, she underwent a CT angiography of the chest (Figure 1) which demonstrated no pulmonary emboli but revealed patchy bilateral peripheral and basilar ground-glass opacities (Figure 2).

The patient reported compliance with warfarin, and her international normalized ratio (INR) was 2.4 on presentation. She underwent bronchoscopy which showed increasingly blood-tinged return from serial aliquots obtained from her right middle lobe. Cytology was negative and infectious workup was negative except for the growth of a hyaline mold which was attributed to contamination. However, empiric antifungal therapy was initiated given the need for immunosuppression as a treatment for diffuse alveolar hemorrhage.

She was treated with methylprednisolone 500 mg IV daily for three days followed by a prolonged steroid taper. Due to her persistent symptoms, oxygen requirements, and prolonged hospital stay, she was started on rituximab 1 g IV in the hospital. Warfarin was stopped on admission, and she was treated with unfractionated heparin after improvement in hemoptysis. After her oxygen requirements improved to baseline, she was discharged home on warfarin and completed three more doses of rituximab outpatient for a total of four doses. Her antiphospholipid laboratories after two doses of rituximab showed elevated anticardiolipin IgG (>112.0), elevated β2-glycoprotein I IgG (>112.0), and the presence of lupus anticoagulant.

Approximately six months later, she presented again with recurrent hemoptysis and hypoxic respiratory failure requiring bi-level positive airway pressure and high-flow oxygen. CT chest with pulmonary angiogram showed no pulmonary embolism but diffuse bilateral ground-glass opacities and consolidations, more confluent at the bases. She underwent a repeat bronchoscopy with bloody fluid, consistent with diffuse alveolar hemorrhage. Infectious workup was negative with no growth on cultures. INR on this presentation was 2.8. Her anticardiolipin IgG was >112, anti-β2-glycoprotein I IgG was >112, and lupus anticoagulant was present. Warfarin was stopped and she was managed with unfractionated heparin. She was started on methylprednisolone 500 mg IV daily for three days followed by a prolonged taper. Due to her critical pulmonary status, recurrent diffuse alveolar hemorrhage refractory to steroids and rituximab, and persistently elevated antiphospholipid antibodies, she was treated with cyclophosphamide 600 mg/m^2^ IV. She improved gradually and did not require invasive mechanical ventilation. She was ultimately discharged on warfarin, steroid taper, and a plan for the continuation of cyclophosphamide outpatient every month. After four months of follow-up, she is currently clinically stable on 2 L of oxygen at rest and 3 L with activity. She remains free of bleeding.

## Discussion

Diffuse alveolar hemorrhage can result from a variety of conditions, including vasculitis, rheumatic diseases, infections, drugs, or toxins [8]. Initial evaluation requires a thorough history and physical examination, targeted serologic analysis to exclude rheumatologic causes, thorough infectious workup, and bronchoscopy with bronchoalveolar lavage [8]. CT findings for diffuse alveolar hemorrhage are typically described as patchy ground-glass or airspace opacities with lower lobes more commonly involved; progression to consolidative changes may be seen in later days [9]. The patient in our case report had underlying APS, and each time she presented with hemoptysis, dyspnea, and hypoxia, she underwent CT scans of the chest showing bilateral ground-glass opacities, multiple bronchoscopies with bronchoalveolar lavage showing blood in fluid, and negative rheumatologic and infectious workup. This was consistent with diffuse alveolar hemorrhage in the setting of her APS.

Management of diffuse alveolar hemorrhage in antiphospholipid syndrome

Diffuse alveolar hemorrhage with underlying APS has a high mortality rate with one study reporting approximately 21% mortality rate among all cases [10]. Treatment involves achieving hemodynamic stability and maintaining ventilation and oxygenation, hemostasis, and treatment of underlying APS with immunosuppression [11]. Early initiation of high-dose corticosteroids (at least 500 mg methylprednisolone IV for three to five days) is recommended with a prolonged taper (prednisone) to combat the inflammatory response, although steroids alone may not be enough to achieve or maintain response in some patients [11].

Remission rates are improved with the addition of rituximab or cyclophosphamide in addition to high-dose steroids, especially in cases refractory to steroids alone [11-13]. Rituximab is a monoclonal antibody against CD20 and may have a role in decreasing antiphospholipid antibodies, such as anticardiolipin IgG, leading to improved response rates [14]. Cyclophosphamide is an alkylating agent with high immunosuppressive activity and likely plays a role in the treatment of diffuse alveolar hemorrhage related to APS through lymphocyte depletion [15].

Additional therapies such as intravenous immunoglobulin (IVIG), mycophenolate mofetil, hydroxychloroquine, azathioprine, and plasmapheresis may be considered, although response rates may be lower compared to rituximab or cyclophosphamide [11]. Although its exact mechanism is unclear, IVIG may be effective in the treatment of APS through the inactivation of B-cell clones and the neutralization of autoantibodies such as anticardiolipin [16]. Hydroxychloroquine may have a role in decreasing the recurrence of thrombosis in APS, although its effects on diffuse alveolar hemorrhage are unclear [17]. Mycophenolate mofetil and azathioprine decrease autoantibody production through B-cell and T-cell suppression [18].

Due to limited effective treatment options and high mortality rates in APS-associated diffuse alveolar hemorrhage, additional therapies are needed. Canaud et al. recently demonstrated IgG antibodies in APS activating mammalian target of rapamycin (mTOR) through the phosphatidylinositol 3-kinase-AKT pathway and beneficial effects of sirolimus in renal transplant patients with APS [19]. The effectiveness of targeting mTOR in complications from APS affecting other organs requires further evaluation but may be a potential treatment option in the future. Other treatment options are currently under study in preclinical models including complement inhibitors and synthetic peptides modulating β2-glycoprotein I binding [20].

In addition to the treatment of underlying APS, maintaining hemodynamic stability and achieving early hemostasis are important components of managing diffuse alveolar hemorrhage. Patients may require treatment within intensive care units, especially when they require high-flow oxygen, noninvasive positive-pressure ventilation, or invasive mechanical ventilatory support. Patients on anticoagulation (e.g., warfarin) often require discontinuation of anticoagulation during the acute hemorrhage phase but their anticoagulation should be resumed as soon as bleeding is controlled [11]. Additional interventions, such as the administration of recombinant factor VIIa, have been utilized for hemostasis but are not currently standard of care [12]. Diagnosis and management of diffuse alveolar hemorrhage in APS can be challenging and requires a multidisciplinary approach along with vigilant monitoring for recurrence.

## Conclusions

Diffuse alveolar hemorrhage is a rare but life-threatening complication of APS that requires prompt diagnosis and management. This case describes the importance of early treatment with corticosteroids and immunosuppressive agents such as rituximab and cyclophosphamide.
